# Small Molecule-Assisted, Line-Independent Maintenance of Human Pluripotent Stem Cells in Defined Conditions

**DOI:** 10.1371/journal.pone.0041958

**Published:** 2012-07-30

**Authors:** Stefan Frank, Miao Zhang, Hans R. Schöler, Boris Greber

**Affiliations:** 1 Max Planck Institute for Molecular Biomedicine, Münster, Germany; 2 University of Münster, Medical Faculty, Münster, Germany; 3 Chemical Genomics Centre of the Max Planck Society, Dortmund, Germany; National University of Singapore, Singapore

## Abstract

Human pluripotent stem cells (hPSCs) are conventionally grown in a mouse feeder cell-dependent manner. Chemically defined culture conditions are, however, desirable not only for potential medically oriented applications but also for investigating mechanisms of self-renewal and differentiation. In light of the rather high complexity and cost of existing defined hPSC culture systems, we have systematically evaluated over 20 potential media ingredients. Only components that reproducibly gave beneficial effects were ultimately combined to yield a simple and cost-effective formulation termed FTDA. This xeno-free medium is based on mimicking self-renewal factor activities present in mouse embryonic fibroblast-conditioned medium, at minimal dosages. Additionally, small molecule inhibitors of BMP and WNT signaling served to specifically suppress typical types of spontaneous differentiation seen in hPSC cultures. FTDA medium was suitable for the generation of human induced pluripotent stem cells and enabled robust long-term maintenance of diverse hPSC lines including hard-to-grow ones. Comparisons with existing defined media suggested reduced spontaneous differentiation rates in FTDA. Our results imply that using supportive factors at minimal concentrations may still promote robust self-renewal and preserve pluripotency of hPSCs.

## Introduction

Human embryonic stem cells (hESCs) were first derived and maintained on feeder layers of mitotically inactivated mouse embryonic fibroblasts (MEFs) in fetal calf serum-containing media [1], [2]. Driven by their potential use in future regenerative medicine, however, considerable efforts have been made to develop feeder-free and chemically defined hESC culture systems. A first step into this direction was pioneering work by Amit et al. [3] who showed that serum could be substituted by the more defined but proprietary serum replacement (KSR, Invitrogen) and fibroblast growth factor 2 (FGF2). Subsequently, Xu et al. showed that Amit's FGF2-containing medium could be used to produce conditioned medium for reliable feeder-free maintenance of hESCs, by incubating it on confluent layers of MEFs [4].

Later, it was shown that one function of FGF2 in this system is to sustain self-renewal of hESCs in an indirect manner - FGF2 changes gene expression in MEFs to turn these into supportive feeder layers [5]: FGF2 stimulation of MEFs leads to secretion of TGFβ1 and Activin A, as well as of Gremlin, an antagonist of bone morphogenic protein (BMP) signaling [5]. Indeed, TGFβ1 and Activin A have been shown to support self-renewal of hESCs, in cooperation with FGF2 [6], [7], [8]. In contrast, BMP signaling is generally thought to promote differentiation of hESCs [9], [10]. Hence, recombinant Gremlin contained in MEF-conditioned medium will serve to counteract spontaneous differentiation.

Based on these and other findings, a number of - largely or fully defined - hESC media formulations have been developed that can roughly be categorized into: (i) media that mostly rely on FGF2 supplementation [11], [12], [13], [14], [15], [16], [17], [18], (ii) media that contain high dosages of FGF2 and a BMP antagonist [19], [20], (iii) media that are based on adding FGF2 together with TGFβ1 [7], [21], [22], and (iv) media based on FGF2 plus Activin A [8], [23]. We wondered whether combining these activities - as they are apparently all present in MEF-conditioned medium [5] - would have additive positive effects on maintaining the undifferentiated state of hESCs.

A recent comparison of several defined hESC media suggested that essentially only two proprietary commercial media allowed for robust expansion of many different hESC lines [24]. However, for more and more widely used procedures such as expansion and characterization of clonal lines of induced pluripotent stem cells (hiPSCs) [25], [26], costs of culture media become an increasingly relevant factor for many laboratories. Moreover, functional studies of self-renewal and induction of differentiation in hPSCs require not only the use of defined media but also a disclosed media composition that can be adapted to specific needs. Along these lines, several published media contain growth factors the effects of which have not been rigorously tested. Yet other formulations contain growth factors at superphysiological concentrations, which may be necessary to balance adverse effects of other non-optimized components in those media.

We therefore sought to define what may be a minimal defined medium for hPSCs. Our strategy involved starting off with a simple published medium and optimizing it in a stepwise manner. We required (i) that only factors/ingredients shall be included that do have reproducible positive effects on hPSC maintenance, (ii) that concentrations of growth factors and other components should be optimized - i.e. minimized - whenever appropriate, and (iii) that the medium must enable maintenance of different independent hPSC lines - hESCs and hiPSCs. Here, we describe FTDA, a minimal defined hPSC media formulation that appears to largely overcome many of the above mentioned shortcomings.

## Results

### Dorsomorphin is a Useful Media Additive Preventing Extraembryonic Differentiation

**Figure 1:**
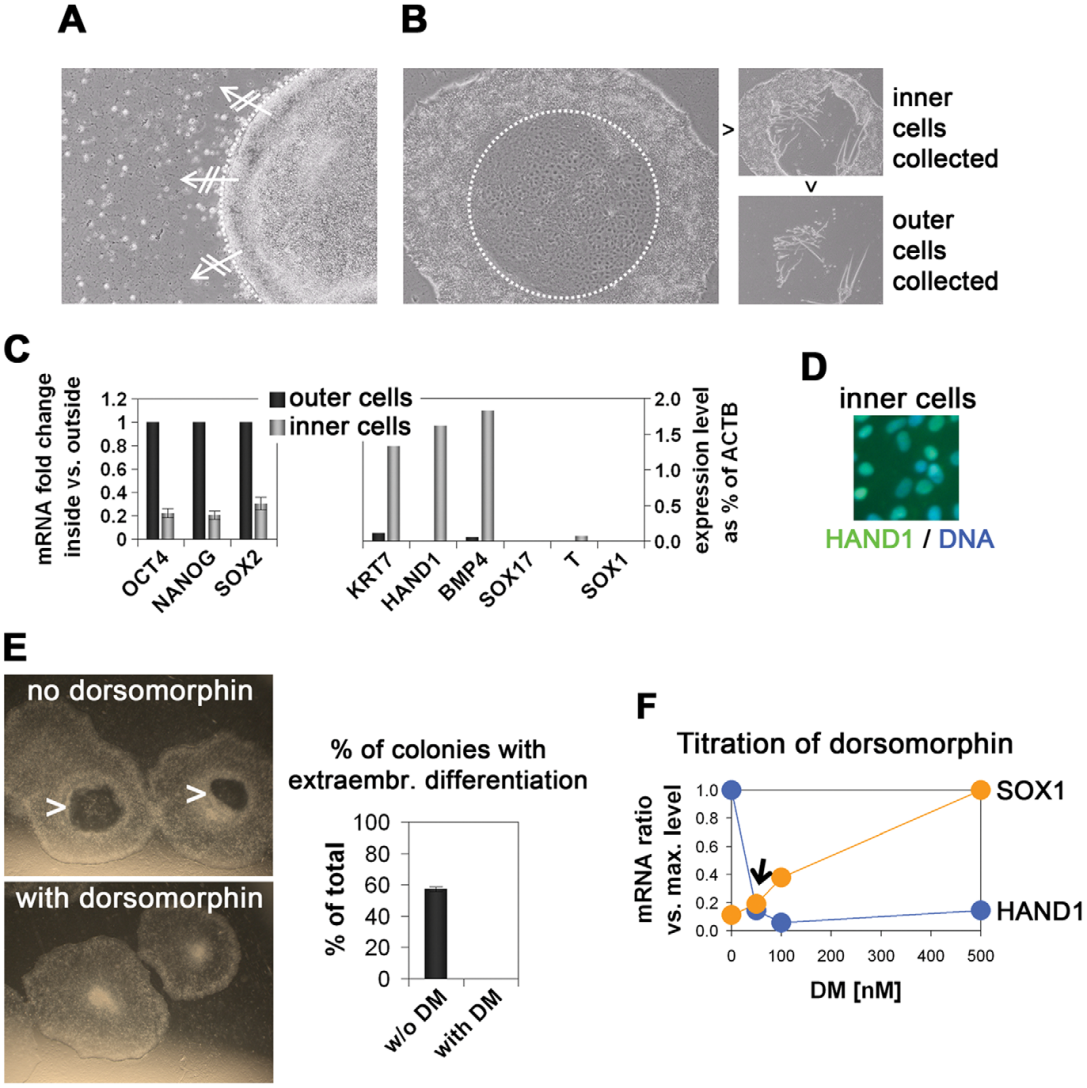
Dorsomorphin prevents spontaneous TE-like differentiation in chemically defined hESC medium. (**A**) Colonies in N2B27+ FGF2 fail to expand well after several passages (line HuES6). (**B**) Typical spontaneous differentiation formed in centers of colonies. Representative differentiated and undifferentiated parts were collected for further analysis (right panels). (**C**) Real-time RT-PCR analysis of samples in (B). (**D**) Immunocytochemistry confirms that inner differentiated cells stain positive for HAND1. (**E**) Dorsomorphin addition to N2B27 completely prevents TE-like differentiation in routine culture (line H7). (**F**) Titration of optimal dorsomorphin dosage. At 50 nM, both TE-like and neural differentiation rates were minimal.

As a starting point, we employed a simple chemically defined medium that contains DMEM/F12 with N2 and B27 supplements, 0.05% of additional bovine serum albumin (BSA), 20 ng/ml FGF2, L-glutamine, non-essential amino acids, and β-mercaptoethanol [14]. Transferring hESCs from MEF-conditioned medium into N2B27+ FGF2 indeed resulted in hESC colonies with overall undifferentiated morphology. Upon serially passaging of the cells, however, many colonies started to develop particularly sharp edges, which interfered with further lateral expansion in our hands (Figure 1A). Moreover, we could observe a change of morphology that always occurred in the center of the colonies. These areas appeared to contain large syncytial cells (Figure 1B). In order to determine the type of this differentiation, we selectively isolated the inner cells and the outer cells and analyzed the expression of different marker genes. This revealed that the inner cells showed strongly reduced levels of the pluripotency genes *OCT4*, *SOX2*, and *NANOG* with concomitant upregulation of the differentiation markers *KRT7*, *HAND1*, and *BMP4*, likely indicating spontaneous extraembryonic differentiation driven by autocrine BMP signaling (Figure 1C, D) [10]. We therefore tested whether dorsomorphin (DM), a small molecule inhibitor of the BMP pathway [27], could prevent this type of differentiation. Indeed, addition of DM to N2B27 completely abolished the formation of the doughnut-shaped colonies that accounted for as many as 60% of all colonies with some hESC lines (Figure 1E, line H7). Since, on the other hand, BMP inhibition has also been shown to promote neural induction of hESCs [28], we tested different concentrations between 10 to 500 nM of DM and measured the expression levels of *HAND1* and *SOX1* as markers for extraembryonic and neuroectodermal differentiation, respectively. This revealed that the optimal concentration at which the levels of both these markers were minimal was as low as 50 nM (Figure 1F). Hence, adding low amounts of DM to defined hESC media effectively prevents extraembryonic differentiation without concomitantly promoting neural fate.

### Improved Support of Self-renewal by Combining Low Dosages of FGF2, TGFβ1, and Activin A

**Figure 2:**
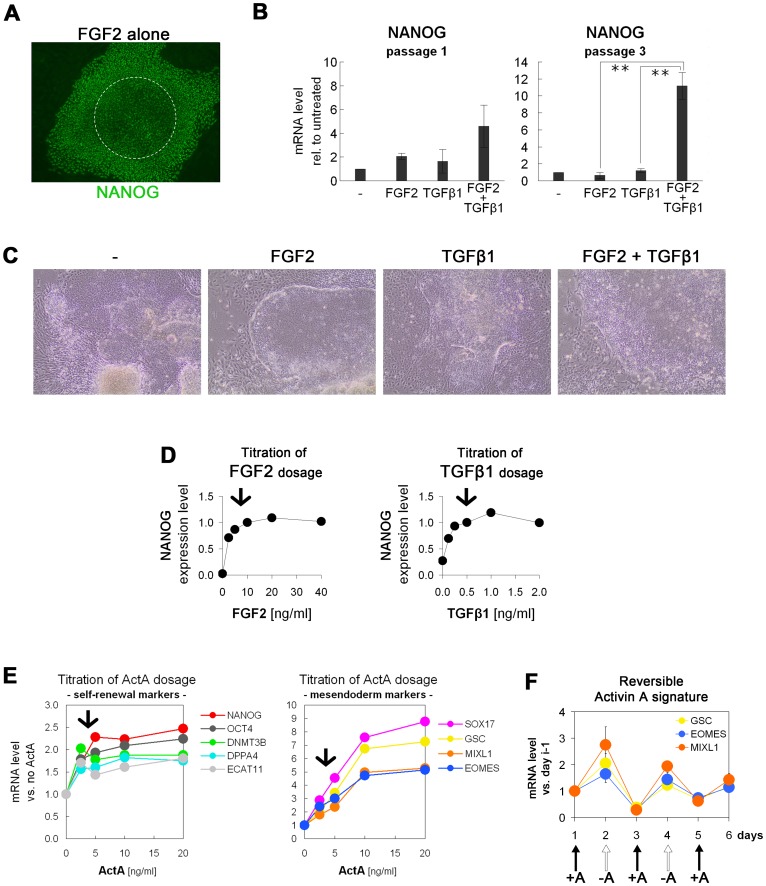
FGF2, TGFβ1, and Activin A cooperatively sustain self-renewal in hESCs. (**A**) Colonies in N2B27+ FGF2 with or without DM typically exhibit weak NANOG staining in their centres. (**B**) RT-qPCR analysis showing that FGF2 and TGFβ1 sustain *NANOG* in a cooperative or synergistic manner (passage 1: n = 2, passage 3: n = 3, all in presence of DM). (**C**) Typical morphology at passage 2 showing that FGF2 or TGFβ1 alone fail to robustly sustain self-renewal. When applied in combination, however, larger colonies with reduced rates of differentiation are obtained. (**D**) Titration of FGF2 and TGFβ1 dosage using the *NANOG* expression level as a readout, in presence of DM. Arrows mark concentrations chosen for further investigation. (**E**) RT-qPCR analysis showing that additional supplementation with Activin A further enhances the expression of self-renewal genes in hESCs (left chart, n = 5). Note that a dosage of 2.5 to 5 ng/ml was sufficient to achieve near-saturating expression levels. In contrast, the induction of mesendodermal differentiation genes at this concentration of Activin A was still moderate (right chart, n = 5, in presence of FGF2, TGFβ1, and DM over three passages). (**F**) Analysis of the mesendodermal gene induction signature by Activin A (n = 3, in presence of FGF2, TGFβ1, and DM). Arrows mark the supplementation with Activin A. Note that the Activin-induced upregulation of mesendodermal markers *EOMES, GSC,* and *MIXL1* is fully reversible. Data in this figure: Line HuES6.

Continuously growing hESCs in FGF2 and DM, however, still only gave rise to relatively small colonies, as compared to cells in MEF-conditioned medium, and these also had reduced levels of NANOG in their centers (Figure 2A). We therefore investigated whether addition of TGFβ1 cooperated with FGF2 in sustaining *NANOG* in the cultures. Indeed, combining FGF2 with TGFβ1 sustained *NANOG* expression in a synergistic manner over several passages (Figure 2B). Interestingly, applying both FGF2 and TGFβ1 in combination also yielded larger colonies of undifferentiated hESCs, whereas each factor alone was not able to produce this morphology (Figure 2C). Using *NANOG* expression as a readout to titrate the concentrations of FGF2 and TGFβ1 revealed that near-saturating levels were already obtained at rather moderate dosages of 5 to 10 and 0.5 ng/ml, respectively (Figure 2D). Moreover, we found that Activin A, which is activating the same signaling pathway as TGFβ1, but via different receptors [29], could further increase the expression of self-renewal associated genes around 2-fold. Since, however, Activin A is also a potent inducer of mesendodermal differentiation in hESCs [30], we also investigated the induction of mesendodermal markers in response to Activin A. Titrating Activin A with regards to enhancing the expression of self-renewal genes but only moderately causing induction of mesendodermal differentiation genes, we found - as a compromise - that 2.5 to 5 ng/ml of Activin A appeared to be the optimal concentration range (Figure 2E). Since even moderate levels of mesendodermal differentiation may be a concern when routinely culturing hPSCs, we performed an Activin A "spike-in" experiment in which Activin was only added every other day throughout a given passage. This revealed that the induction of mesendodermal genes by Activin A is a fully reversible phenomenon and hence suggested that routine addition of low dosages shall not present a point of concern (Figure 2F). Taken together, these data show that combining moderate dosages of FGF2, TGFβ1, and Activin A - the major relevant factors contained in MEF-conditioned medium [5] - results in cooperative support of hPSC-specific gene expression.

### Optimization of Accessory Media Ingredients and Global Expression Profile

**Figure 3:**
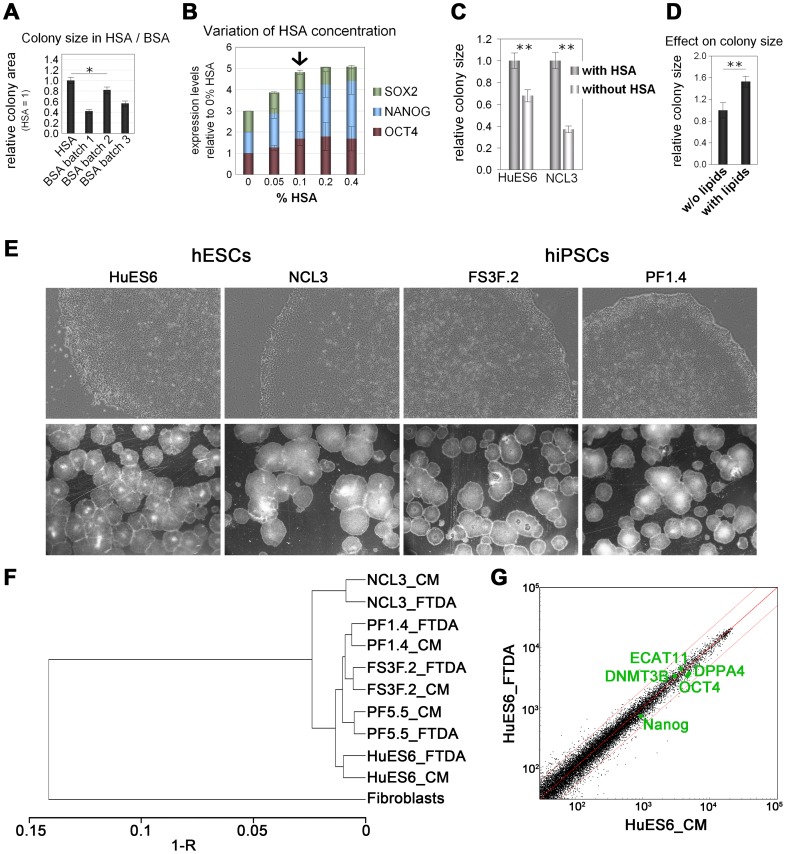
Optimization of accessory media ingredients. (**A**) HSA supplementation caused superior colony expansion compared to BSA. (**B**) 0.1% HSA was sufficient for obtaining saturated expression levels of *OCT4*, *NANOG*, and *SOX2*. (**C**) HSA supplementation promoted improved colony expansion as compared to albumin-free conditions. (**D**) Addition of defined lipids enabled improved colony expansion with most lines (NCL3 hESCs, n = 2). (**E**) Optimized FTDA medium enabled robust maintenance of arbitrarily chosen hES and hiPS cell lines over multiple passages, with minimal spontaneous differentiation. Top: Representative phase contrast morphology. Bottom: Stereo microscopic view. (**F**) Microarray cluster analysis of several hPSC lines cultured in MEF-CM and FTDA as well as human fibroblasts demonstrated clustering of individual lines independent of the medium used. (**G**) Global gene expression comparison of HuES6 hESCs cultivated in MEF-CM and FTDA showed highly similar expression patterns for most genes, including pluripotency markers.

We next sought to optimize accessory media components. Our aim was to only include ingredients that reproducibly enhance the growth and self-renewal of hPSCs but to omit components that do not. This was based on systematically testing the addition/omission of individual components over several passages and analyzing effects by colony size quantification and/or marker gene expression analysis. Replacing N2 supplement by simpler, commercially available mixtures of insulin, transferrin, and selenite (ITS) as well as omitting the B27 supplement yielded the same compact undifferentiated morphology and had no negative effects on colony size (Table 1 and data not shown). To eliminate xenogeneic media components, we compared human serum albumin (HSA) supplementation with BSA. Interestingly, cells in HSA-containing medium always produced larger colonies than in media containing BSA, irrespective of the BSA batch tested (Figure 3A). Titration of HSA revealed that near-maximum levels of the core pluripotency factors *OCT4*, *SOX2*, and *NANOG* were already obtained at a rather moderate concentration of 0.1% (Figure 3B). It has recently been suggested that albumin is dispensable for the cultivation of hPSCs [22]. In our hands, however, removal of HSA always compromised colony expansion (Figure 3B, C). Similarly, testing effects of synthetic chemical polymers revealed that they could not mimic the beneficial effects of HSA (Table 1). Omission of β-mercaptoethanol and non-essential amino acids, which are both frequently included in hESC media, did not impair long-term culture of hPSCs. Rather, cells grown without β-mercaptoethanol performed better, confirming data by Chen et al. [22] (data not shown). Finally, supplementing the medium with defined lipid mixtures yielded significantly larger colonies (Figure 3D). A list of all tested substances is given in Table 1. The final optimized minimal medium - termed FTDA - consisted of only several components: DMEM/F12 with L-glutamine, ITS, HSA, defined lipids, FGF2, TGFβ1, DM, and Activin A.

**Table 1:** Summary of media ingredients tested.

	Tested parameter	Results/remarks
*Included*		
	**DMEM/F12** with L-glutamine	Superior to conventional DMEM; L-glutamine strictly required for cell survival
1x	**ITS**	Insulin strictly required for cell survival; transferrin and selenium not independently tested
0.1%	**HSA**	Larger and more robust colonies than with BSA or without any albumin
1x	**Lipid mix**	Chemically defined, yields somewhat more compact and larger colonies
5–10^a^ ng/ml	**FGF2**	Crucial self-renewal factor
0.5 ng/ml	**TGFβ1**	Cooperates with FGF2 to enhance *NANOG* expression
50 nM	**Dorsomorphin**	Prevents extraembryonic differentiation in centres of colonies
2.5–5^a^ ng/ml	**Activin A**	Cooperates with FGF2 and TGFβ1 to enhance *NANOG* expression
*Optional*		
2 µM	IWP-2	Prevents mesendoderm-like differentiation at edges of colonies, but not required for most lines
250 µM	Ascorbate	No consistent effect under albumin-containing conditions, may be beneficial for some lines
	Penicillin & streptomycin	To prevent bacterial contamination
*Tested but excluded*		
	N2 supplement	Replaced by ITS without loss of performance
	B27 supplement	Not required
	BSA	Smaller colonies than with HSA, significant batch-to-batch variation
	Albumax	Lipid-rich albumin component of KSR; yielded rather loose colonies
	HEPES	No measurable positive effect when included in DMEM/F12
	Dextran/PVA^b^/PVP^c^	Tested to replace HSA, no clear improvement of cell growth in longer term
	Fibronectin (soluble)	Tested to improve compactness, no effect
	β-mercaptoethanol/thioglycerol	Slightly toxic, esp. under albumin-free conditions, no measurable positive effect
	Non-essential amino acids	No effect
	Phorbol ester	TPA^d^; tested to potentially replace FGF2, failed to maintain self-renewal beyond P2
	TGFβ2	Similar performance as TGFβ1
	TGFβ3	Less potent than TGFβ1 at equimolar concentrations
	CHIR99021	Counterproductive, induces mesodermal gene expression
	human LIF	No detectable effect

a Batch-dependent.

b Polyvinyl alcohol.

c Polyvinylpyrrolidone.

d 12-O-tetradecanoylphorbol-13-acetate.

Interestingly, although the medium was optimized employing only two hPSC lines, FTDA supported the undifferentiated growth of any hESC or hiPSC line tested (8 in total). Typically, the cells formed large and homogeneous colonies in FTDA (Figure 3E). One particular hiPSC line ("iPS 1"), however, sometimes showed a distinct outgrowth of differentiated cells when cultivated in FTDA (Figure S1A, left). We isolated the outgrowing cells and compared their gene expression to cells from the undifferentiated parts of the colonies and found an upregulation of mesendodermal markers (Figure S1B). To overcome this differentiation, we applied a small molecule inhibiting the production of autocrine WNT proteins, IWP-2 [31]. A concentration of 2 µM was sufficient to completely block the mesendodermal outgrowths in line hiPS 1 (Figure S1A, right). Gene expression analysis revealed a strong downregulation of the basal expression levels of mesendodermal genes in the presence of IWP-2 (Figure S1C, D). In contrast, pluripotency genes were not affected by IWP-2 treatment (Figure S1E). These data suggest that IWP-2 may be a useful small molecule to inhibit spontaneous mesendodermal differentiation in hPSC cultures, if necessary (Table 1).

To confirm at a global scale that FTDA supports self-renewal of diverse hPSC lines, gene expression profiles of 5 hPSC lines cultivated in FTDA as well as in MEF-CM were recorded by means of microarrays. This revealed that on the one hand, all hPSC-lines clustered closely with one another. On the other hand, cell-line specific characteristics appeared to be preserved, because line-to-line differences were overall stronger than media-induced differences (Figure 3F). Scatter plots showed that with a given cell line, global gene expression profiles of cells in MEF-CM versus FTDA were highly similar with virtually unaltered levels of pluripotency markers (Figure 3G). These observations suggest that global gene expression in FTDA indeed resembles that in MEF-CM.

### Long-term Self-renewal and Pluripotency in FTDA

**Figure 4:**
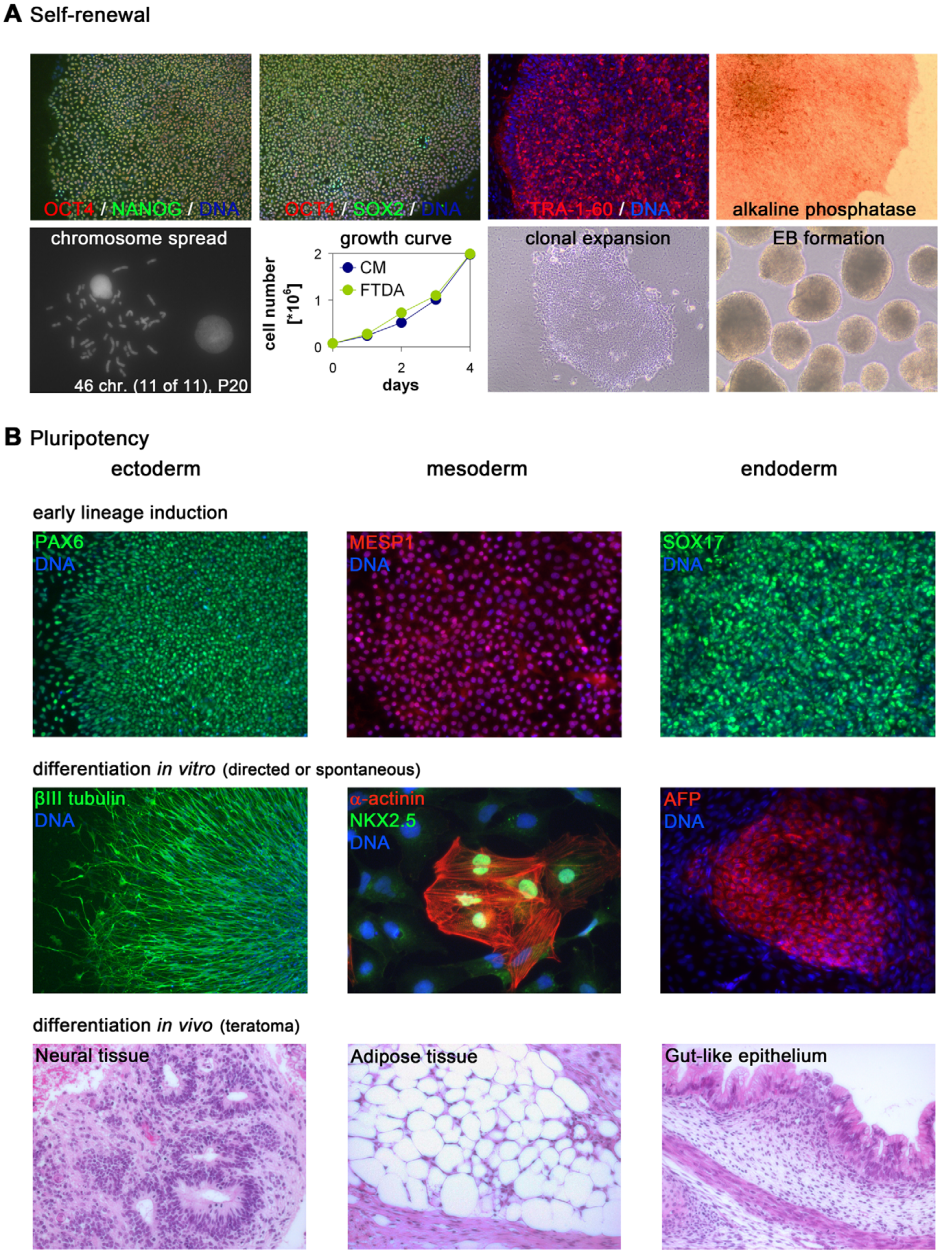
Validation of optimized FTDA medium. (**A**) hESCs grown in FTDA medium for more than 20 passages exhibited robust expression of self-renewal markers (top row), stable karyotype, and rapid growth rates (bottom left). Bottom right: FTDA allowed clonal expansion from single cells replated in the presence of Y27632 as well as effective formation of embryoid bodies. (**B**) hES cells grown in FTDA for multiple passages could be induced to form early neuroectodermal, mesodermal, or endodermal precursors, using the same basal medium but different growth factors (top row). Using directed or spontaneous differentiation protocols, cells gave rise to terminally differentiated cell types of all three germ layers (middle row). Bottom: H&E stained teratoma sections formed by HuES6 cells grown for more than 20 passages in the defined medium.

Next, we sought to assess the long-term maintenance of PSC features under FTDA conditions. To this end, hESC line HuES6 was maintained in FTDA for more than 20 passages and characterized as follows. hESC colonies showed homogenous expression of the pluripotency factors *OCT4*, *NANOG*, and *SOX2* throughout the colonies, along with immunoreactivity for TRA-1-60. The cells showed alkaline phosphatase activity and maintained a stable number of chromosomes. Growth rate analysis showed no difference between FTDA and MEF-conditioned medium and flow cytometry following intracellular staining revealed 95% of OCT4-positive cells in FTDA conditions. Cells in FTDA could be clonally expanded after a single-cell seeding (with an efficiency of ∼5% upon addition of 10 µM ROCK inhibitor Y27632), like it is for instance required in genetic selection procedures. Furthermore, embryoid bodies could readily be generated in FTDA, which is a prerequisite for many differentiation protocols (Figure 4A, S2A, B).

Upon altering the composition of growth factors and small molecules, but maintaining the same basal media components, we were able to induce early lineage specification along all three germ layers (Figure 4B, top). Application of extended in-vitro differentiation protocols enabled us to derive terminally committed cells of ectodermal (neurons), mesodermal (cardiomyocytes), and endodermal (fetal liver-like cells) origin (Figure 4B, middle). By injection of FTDA-cultivated cells into immunodeficient mice, we obtained teratomas containing tissues from all three germ layers (Figure 4B, bottom, S2C). Taken together, these data show that hPSCs grown in FTDA for multiple passages retain their defining characteristics of self-renewal capability and pluripotency.

### Direct Reprogramming and Media Comparison

**Figure 5:**
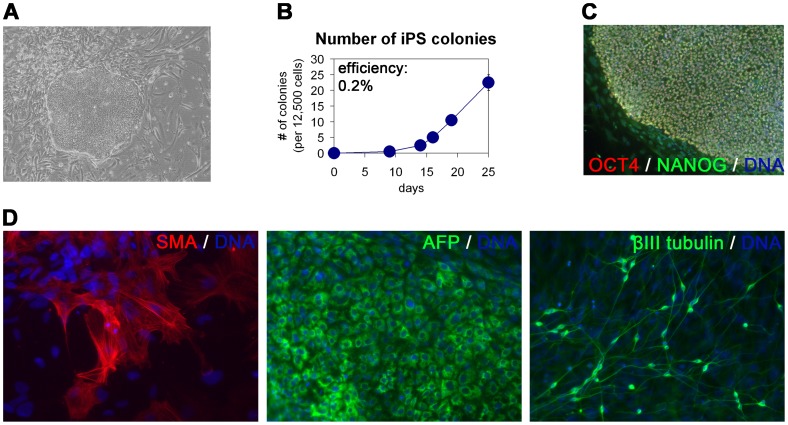
Direct reprogramming in FTDA. (**A**) FTDA allowed derivation of hiPS colonies after viral transduction of adult dermal fibroblasts with OCT4, SOX2, KLF4 and c-MYC. (**B**) The number of hiPS colonies increased over time, reaching an average of 24 per 12,500 seeded cells (0.2%). (**C**) Emerging hiPS colonies could be picked and expanded in FTDA and homogenously stained positive for pluripotency markers *OCT4* and *NANOG*. (**D**) Spontaneous in-vitro differentiation of hiPSCs derived under FTDA conditions yielded cell types representative of all 3 germ layers.

Beyond sustaining pluripotency, we also asked if FTDA was compatible with the induction of pluripotency during transcription factor-mediated reprogramming of adult fibroblasts [25], [26]. Indeed, in a typical setting, the first hiPS colonies appeared 10–14 days after viral transduction (Figure 5A), and the overall reprogramming efficiency was at 0.2%, i.e. comparable to published data (Figure 5B) [25], [26]. Nascent hiPSC colonies could be readily picked and expanded in FTDA and stained positive for *OCT4* and *NANOG,* as expected (Figure 5C). Upon cultivation as embryoid bodies and subsequent plating into differentiation media, hiPSCs derived in FTDA readily differentiated into cells representative of all three germ layers, demonstrating pluripotency of the obtained cells (Figure 5D). Hence, FTDA was compatible with standard direct reprogramming protocols.

**Figure 6:**
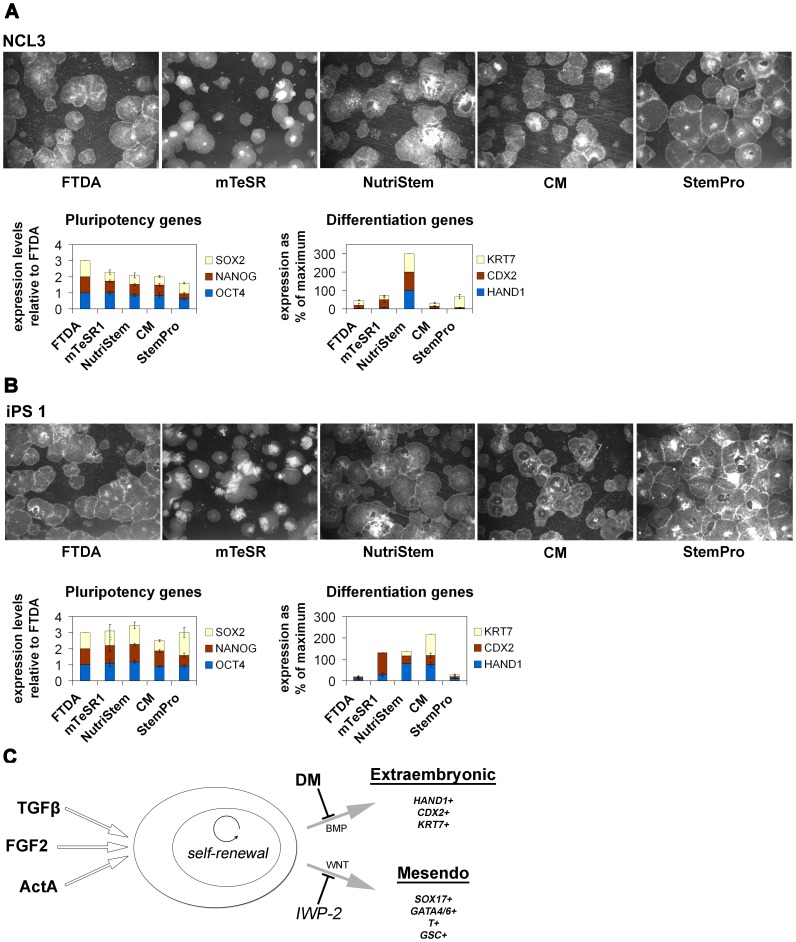
Media comparison and model of FTDA mechanism. (**A**) hESC line NCL3 and (**B**) iPS line 1 were cultured for 3 passages in FTDA, in MEF-CM, and in proprietary media. All media sustained pluripotency, but differentiation tended to be increased in MEF-CM and some proprietary media, compared to FTDA. Top: Stereo microscopic view. Bottom: Gene expression analysis relative to FTDA (n = 3). (**C**) Model of our understanding of the pluripotency-sustaining mechanism of FTDA. FGF2, TGFβ1 and Activin A cooperatively support self-renewal, whereas DM inhibits spontaneous extraembryonic differentiation. IWP-2 may additionally be used if cells tend to spontaneously differentiate into mesendoderm.

In order to assess how FTDA compares with commercially available hPSC media, we cultured a hESC line (NCL3) and a more difficult-to-grow hiPSC line (iPS 1) for 3 passages in FTDA, in MEF-CM, as well as in proprietary NutriStem®, StemPro®, and mTeSR®1 media. We were able to maintain both lines in all the media, whereas there were differences with regards to spontaneous differentiation rates. While overall high percentages of undifferentiated colonies were found in all conditions, the number of differentiated areas appeared to be lowest in FTDA. The extent of spontaneous differentiation – mostly with extraembryonic morphology – was also dependent on the hPSC line, being more prominent in line iPS 1 than in NCL3 hESCs (Figure 6A, B, top). Gene expression analysis confirmed these observations in that the expression levels of pluripotency markers in bulk cultures were comparable or lower in the other media, whereas expression levels of (extraembryonic) differentiation marker genes were comparable or higher in the other media, as compared to FTDA. As expected from the morphologies, this effect was again more pronounced in the iPS 1 cells (Figure 6A, B, bottom). Similar results were obtained with yet another defined medium, the recently published E8 formulation [22], again showing less spontaneous differentiation in FTDA (data not shown). Overall, these data suggest that FTDA promotes robust hPSC growth with rather low rates of spontaneous differentiation even in harder-to-grow lines.

## Discussion

In 2001, Xu et al. developed a widely used protocol for feeder-free culture of hESCs, based on conditioning conventional hESC medium by embryonic fibroblasts [4]. Later, it was found that the relevant secreted factors most likely mediating the hESC-supportive activity in MEF-conditioned medium were members of the TGFβ family of ligands, namely TGFβ1, Activin A, and the BMP antagonist Gremlin [5], [32]. This conclusion was based on previous findings by several groups showing that FGF2 cooperates with TGFβ1 or Activin A or with BMP antagonists in sustaining self-renewal of hESCs [6], [7], [8], [19], [20], [21]. Here, we show that combining all these factors/activities - FGF2, TGFβ1, Activin A, and anti-BMP - under chemically defined conditions enables particularly robust maintenance of various hPSCs lines without a loss of pluripotency. According to our data, FGF2, TGFβ1 and Activin A induce self-renewal genes in a cooperative - i.e. non-redundant - manner, which appeared to make this combination superior to using only one or two factors. Moreover, some previously published media include growth factors that have not unequivocally been shown to support hESC maintenance or contain factors at super-physiological concentrations. In addition, some of these, such as Activin A, are ambivalent factors that may also induce differentiation at higher dosages [14], [30], [32]. We therefore think that careful titration of growth factors and their subsequent use at rather conservative dosages may be most appropriate.

In addition to actively promoting self-renewal using receptor ligands, spontaneous differentiation could effectively be blocked by small molecules (Figure 6C). Mimicking the effect of BMP antagonists that are present in MEF-CM, we applied dorsomorphin, a small molecule inhibitor of BMP signaling, to prevent spontaneous extraembryonic differentiation. As in case of the growth factors, however, we observed that using DM at a minimal dosage is crucial because at higher concentrations enhanced rates of neural differentiation were frequently obtained. This is in line with the finding that BMP inhibition may indeed also favor neural induction of hPSCs [28], [33] and it once more illustrates that dosages of ambivalent factors in hPSC media need to be well optimized. Moreover, in one hiPSC line, additionally applying a small molecule inhibitor of WNT signaling, IWP-2, allowed us to react to cell line-specific propensity for spontaneous mesendodermal differentiation. Albeit being beneficial in one hPSC line, however, we consider this an optional media component, since long-term culture of that line was not significantly compromised in the absence of IWP-2. Taken together, mimicking activities found in MEF-CM - by combining the three growth factors and DM (FTDA) - robustly sustained pluripotency in various hPSC lines both by actively promoting self-renewal and blocking spontaneous differentiation (Figure 5F).

Beyond optimizing the signaling environment, our testing of other media ingredients also highlights the advantage of optimizing seemingly unimportant ingredients and excluding counterproductive ones. Chen et al. recently took a similar approach to simplify defined hPSC culture conditions, which resulted in the E8 formulation with only 8 main components [22]. FTDA shares some features with E8 in that it, for example, excludes the rather adverse activity of β-mercaptoethanol. However, FTDA requires only 5 to 10% of the FGF2 and 25% of the TGFβ1 concentrations contained in E8, resulting in more cost-efficient culture of hPSCs. Moreover, the E8 conditions do not contain any form of serum or serum proteins. In line with data by Chen et al., we did not actually observe any positive effects associated with bovine serium albumin supplementation. In contrast, however, we found that human serum albumin preparations had a significant positive effect on the expression levels of pluripotency genes as well as on colony expansion. In addition, HSA appeared to counteract spontaneous neural differentiation, which was also the predominant form of differentiation seen with HSA-free E8 medium in our hands (data not shown). It remains to be determined whether it is the human albumin itself or associated molecules carrying these obscure but beneficial activities.

Besides using MEF-CM, many groups employ proprietary media for cultivation of hPSCs. However, these media are usually expensive and their compositions are either not disclosed or rather complex. Nonetheless, in a comparative study it was shown that some of these media reliably support the maintenance of several independent hPSCs lines [24]. Similarly, FTDA was suitable for sustaining self-renewal of all hPSC lines tested, including harder-to-grow ones, with low rates of spontaneous differentiation. Moreover, by modifying the growth factor composition but maintaining its basal ingredients, directed differentiation along all three germ layers could selectively be initiated. This suggests that FTDA not only presents a cost-effective alternative for robust large-scale expansion of hPSCs but also a versatile media platform for studying mechanisms of self-renewal and differentiation.

## Materials and Methods

### Cell Culture

Validated HuES6 and NCL3 hESCs were from the Harvard Office of Technology Development and the UK Stem Cell Bank, respectively, and used below passage 50 [34], [35]. hESCs and hiPSCs (see below) were exclusively grown under feeder-free conditions using 6 and 12-well tissue culture plates coated with 0.0125% (v/v) diluted Matrigel® HC (BD) [36]. The medium used as a starting point for optimization was N2B27 with FGF2 [14]. Products deviating from those described in [14] were confirmed to be rather independent of the supplier: N2B27 contained DMEM/F12 (Invitrogen #21331-020, PAA #E15-012, or Hyclone #SH3012601), 1x N2 supplement (Invitrogen #17502-048 or PAA #F005-004), 1x B27 supplement (Invitrogen #12587-010), 0.05% BSA (Sigma #A1595), 2 mM L-glutamine (PAA #M11-004 - if not contained in DMEM/F12), 1x non-essential amino acids (PAA #M11-003), 0.1 mM β-mercaptoethanol (Invitrogen #31350-010), 1x penicillin/streptomycin (PAA #P11-010), and 20 ng/ml FGF2 (Peprotech #100-18B).

For splitting cells, hPSC colonies were cut into equally sized fragments using a sterile injection needle. After washing once with PBS, the cells were incubated with 2 mg/ml Dispase (Invitrogen #17105-041) for 5–10 min at 37°C and subsequently washed three times with PBS. Cells were then scraped off with a sharp cell scraper and transferred to a 15 ml conical tube. After brief centrifugation (5s at 200g), clumps were resuspended in medium supplemented with 10 µM Y27632 (Abcam #ab120129) and transferred to equilibrated medium in Matrigel®-coated culture dishes.

For stepwise systematic media optimization, cells were grown in the presence or absence of a given factor and monitored under these conditions over a period of at least 3 passages. Towards the end of a given passage, one half of the cells growing in wells of 6-well plates was scraped out and used for RNA isolation, followed by RT-qPCR analysis using panels of self-renewal and early differentiation markers. The other half of a given well was used for splitting and maintaining the cells under the same conditions, followed by repeating the procedure several days later etc. To account for cell line-dependent effects, most optimzation experiments were carried out using two independent hESC lines, HuES6 and NCL3. FTDA medium was compared to the following commercially available media: NutriStem® (Stemgent #01-0005-100), StemPro® (Invitrogen #A10007-01) and mTeSR®1 (Stemcell Technologies #05850). All media were used according to the manufacturers’ protocols. E8 medium was prepared as described in Chen et al. [22], using 100 ng/ml of human FGF2 and 2 ng/ml TGFβ1. Sources of BSA were Sigma #A1595, Invitrogen #A10008-01, and Invitrogen #15260-037.

Optimized FTDA medium was composed of DMEM/F12 (Invitrogen #21331-020), 2 mM L-glutamine (PAA, as not contained in the DMEM/F12 used), 1x ITS (BD Biosciences #354351), 0.1% HSA (Biological Industries #05-720-1B), 1x chemically defined lipids (Invitrogen #11905-031, 1∶100), 5–10 ng/ml FGF2 (Peprotech #100-18B), 0.5 ng/ml TGFβ1 (Peprotech #100-21C), 2.5–5 ng/ml Activin A (R&D #1066-AB-005 or eBioscience #14-8993-62), 50 nM DM (Merck), and 1x penicillin/streptomycin (PAA). Line iPS 1 additionally received 2 µM IWP-2 (Merck). We noticed that Activin A showed marked differences in activity between different manufacturers. We therefore tested several products and, hence after, only used Activin A from R&D or eBioscience. For sustained undifferentiated growth in FTDA, we found it is crucial to feed cells on a daily basis.

For analyzing growth rates, HuES6 hESCs were plated as clumps into replicate wells containing either FTDA or MEF-CM [4]. Every 24 hours, cells from a given well were dissociated with Accutase® and counted. Quantification of colony sizes was done by determining relative colony diameters of at least 20 colonies from a given well and converting the values into relative areas occupied by the colonies. For clonal expansion, cells were split with Accutase, re-seeded at low density in the presence of 10 µM Y27632, and fed with FTDA hence after.

For differentiation into germ layer precursors, hESCs were replated as clumps or single cells in FTDA, followed by treatment with specific growth factors/pharmacological inhibitors, while leaving the other components of the medium unchanged: PAX6-postive neuroectoderm was induced by treating cells for 5 days with 0.5 µM PD0325901 (Axon Medchem), 15 µM SB431542 (Ascent), and 0.5 µM dorsomorphin (DM, Merck) [36]; MESP1-positive mesoderm was induced by treating cells for 2 days with 10 ng/ml FGF2, 5 ng/ml Activin A (eBioscience #14-8993-80), 10 ng/ml BMP4 (R&D), and 5 µM CHIR99021 (Axon Medchem); SOX17-positive endoderm was induced by treatment with 10 ng/ml FGF2, 100 ng/ml Activin A, 10 ng/ml BMP4, and 5 µM CHIR99021 for 1d, followed by 100 ng/ml Activin A for 3 days. For testing the effect of WNT activation in routine culture, CHIR99021 (Axon Medchem) was used at 1–3 µM. Human recombinant LIF (Millipore) was tested at concentrations of up to 50 ng/ml.

For terminal differentiation into neurons, cells growing in FTDA were harvested as for routine passaging, and embryoid bodies formed by plating cell aggregates into suspension culture dishes using basal defined medium (Table 1) with FGF2, DM, and Y27632. For the next 4 days, EBs were treated with 0.5 µM PD0325901, 15 µM SB431542, 0.5 µM DM, and Y27632, then plated out on Matrigel and treated with PD0325901, SB431542, and DM for another 4 days [36]. Beating cardiomyocytes were generated by plating aggregates of FTDA-adapted hESCs in basal defined medium without growth factors (Table 1) onto confluent feeder layers of cardiogenic END2 cells [37]. Medium was changed every 5 days. After 2–3 weeks, beating clusters were manually isolated, digested with trypsin, and replated onto gelatin-coated dishes. Staining for cardiac markers was performed several days later. AFP-positive fetal liver cells were generated by spontaneous EB differentiation: EBs were generated as above, from cells growing long-term in FTDA, and then transferred to DMEM/F12 with 20% FCS. EBs were plated out onto gelatin-coated dishes after 1 week, and stained for AFP 2 weeks later.

### RT-qPCR and Microarray Analysis

Cells were lysed directly in culture dishes, followed by RNA isolation using RNeasy mini kits with on-column DNA digestion (Qiagen). Reverse transcription was carried out using Moloney Murine Leukemia Virus Reverse Transcriptase (USB #78306) and oligo-dT priming with 1 µg of RNA, according to the manufacturer's instructions. The resulting cDNA was diluted with water to then serve as template for real-time PCR using iTaq SYBR Green Supermix with ROX (Bio-RAD) on ABI Prism 7500 instrumentation (∼50 ng of total RNA equivalents per 20 µl reaction). Calculations were performed using the ΔΔCt method with normalization against two housekeeping genes (RPL37A and ACTB). For monitoring effects of media additives in an objective manner, panels of markers indicating self-renewal or differentiation along specific germ layers were employed. Primer sequences underlying the selected data shown in figures are listed in Table S1.

For microarray analysis, 500 ng of purified RNA was used as input. Sample preparation was performed according to the manufacturer’s protocol (Illumina TotalPrep RNA Amplification Kit). Samples were loaded on an Illumina HT-12 v4 BeadChip, hybridized and scanned according to the manufacturer’s protocol. Data analysis was perfomed with Illumina GenomeStudio v2010.3 and Gene Expression Module v1.8.0.

### Immunocytochemistry and Flow Cytometry

Immunocytochemistry was performed as described, using Alexa-488 or -568 conjugated secondary antibodies, as appropriate [38]. Primary antibodies used were: α-T (R&D #AF2085, 1∶100), α-OCT4 (Santa Cruz #sc-5279, 1∶100), α-NANOG (R&D #AF1997, 1∶100), α-SOX2 (Santa Cruz #sc-17320, 1∶50), α-TRA-1-60 (Millipore #90232, 1∶50), α-PAX6 (Covance #PRB-278P, 1∶500), α-MESP1 (Santa Cruz #sc-130461, 1∶25), α-SOX17 (R&D #AF1924, 1∶100), α-beta-III-tubulin (Sigma #T8660, 1∶1000), α-alpha-actinin (Sigma #A7811, 1∶800), α-NKX2.5 (R&D #AF2444, 1∶100), α-AFP (Sigma #A8452, 1∶500), and α-HAND1 (Santa Cruz #sc-9413, 1∶20).

For flow cytometry, 1 well of a 6well-plate with colonies was dissociated with Accutase containing 10 µM ROCK-inhibitor. Cells were then pelleted in a 1.5 ml non-stick microcentrifuge tube for 3 min at 200 g and resuspended in 100 µl PBS. 100 µl 8% paraformaldehyde was added to the suspension and cells were fixed for 10 min at room temperature on a shaking rotator. Staining of the cells was performed similar to immunocytochemistry, but in suspension. Blocking and incubation with antibodies was performed for 30 min at room temperature each. After every step, cells were washed with 1 ml PBS and centrifuged for 3 min at 1000 g. For analysis, stained cells were resuspended in 500 µl PBS and analyzed on Beckman Gallios 10 C 3L instrumentation. Primary antibodies used were: α-OCT4 (Santa Cruz #sc-5279, 1∶50) and mouse-IgG isotype control (Invitrogen, 1∶200).

### Direct Reprogramming

hiPS cells were generated and characterized as in [36], using Yamanaka's OCT4/SOX2/KLF4 or OCT4/SOX2/KLF4/MYC retrovirus cocktails with VSV-G pseudotyping while essentially following Melton's protocol [25], [39]. Briefly, retroviruses were produced in 293T cells transfected with Addgene plasmids 8454, 8449, and 17217–17220 using Fugene 6 (Roche). 12,500 double-infected fibroblasts were replated per well of a 6-well dish on the day after the second infection and fed with FTDA hence after. Foreskin fibroblast-derived FS3F.2 hiPSCs were described in [36]. PF1.4 hiPSCs were generated from a female patient's skin fibroblasts following written informed consent as well as approval by the ethics commission of the University Hospital of Münster, Germany. hiPS 1 cells were a kind gift by Dr. Laugwitz, Technical University of Munich, Germany [40].

### Other Procedures

Karyotypes were evaluated by counting metaphase chomosome spreads stained with Hoechst. Briefly, hPSCs were treated with 100 ng/ml colcemid for 2h, harvested using Accutase, incubated in 37.5 mM KCl for 20 min, and fixed and dropped on glass slides according to standard procedures.

Alkaline phosphatase staining was performed by incubating paraformaldehyde-fixed cells with a 25∶1 mixture of Fast Red chromogen (1 mg/ml, Sigma) and naphthol phosphate solution (0.25%, Sigma) for about 10 min.

For teratoma formation, hPSCs of approximately one well of a 6-well plate were injected subcutaneously into the hind flanks of SCID mice. About 8 weeks later, teratomas were recovered, fixed in Bouin's solution, embedded in paraffin, sectioned (5 µm), and Haematoxylin/Eosin-stained according to standard procedures.

## Supporting Information

Figure S1 **Activation of WNT signaling is counterproductive for hESC self-renewal.** (**A**) Human iPS line 1 showed distinct outgrowths of differentiating cells when cultured in FTDA (left). Application of 2 µM IWP-2 completely blocked this differentiation (right). Representative phase contrast morphology. (**B**) Gene expression analysis of FTDA-mediated outgrowth of cells in iPS line 1. Outgrowing cells showed upregulation of mesendodermal markers, whereas the epithelial cell marker *CDH1* was downregulated compared to the undifferentiated colony centers. (**C**) Selective inhibition of endogenous WNT signaling by adding IWP-2 to FTDA (FTDAI) resulted in strong downregulation of mesendodermal markers (n = 3, iPS line 1). (**D**) Brachyury (T) expression in hESCs depended on WNT signaling. Left: Activation of canonical WNT signaling by continuous exposure to GSK3β inhibitor CHIR99021 (3 µM) induced T expression at the edges of hESC colonies. Middle: In normal FTDA culture, only a small number of T-positive cells could be found at the edges of hESC colonies. Right: Small molecule-inhibition of endogenous WNT signaling (IWP-2) completely removed T-positve cells at the colony periphery. (**E**) IWP-2 had no effect on expression levels of pluripotency markers *OCT4* and *NANOG* (n = 3, iPS line 1).
(TIF)

Figure S2 **Long-term maintenance of pluripotency features in FTDA.** (**A**) Flow cytometry of NCL3 hESCs cultured in FTDA revealed 95% OCT4-positive cells. (**B**) Single cell-plating of hESCs in FTDA required addition of 10 µM ROCK-inhibitor Y27632. Plating efficiency was ∼5% (of all plated hESCs, n = 3). (**C**) H&E stained teratoma sections formed by HuES6 cells grown for more than 20 passages in the defined medium.
(TIF)

Table S1 Real-time PCR primers used in this study.
(XLS)

## References

[1] Thomson JA, Itskovitz-Eldor J, Shapiro SS, Waknitz MA, Swiergiel JJ, et al (1998) Embryonic stem cell lines derived from human blastocysts. Science 282: 1145–1147. 9804556 10.1126/science.282.5391.1145

[2] Reubinoff BE, Pera MF, Fong CY, Trounson A, Bongso A (2000) Embryonic stem cell lines from human blastocysts: somatic differentiation in vitro. Nat Biotechnol 18: 399–404. 10748519 10.1038/74447

[3] Amit M, Carpenter MK, Inokuma MS, Chiu CP, Harris CP, et al (2000) Clonally derived human embryonic stem cell lines maintain pluripotency and proliferative potential for prolonged periods of culture. Dev Biol 227: 271–278. 11071754 10.1006/dbio.2000.9912

[4] Xu C, Inokuma MS, Denham J, Golds K, Kundu P, et al (2001) Feeder-free growth of undifferentiated human embryonic stem cells. Nat Biotechnol 19: 971–974. 11581665 10.1038/nbt1001-971

[5] Greber B, Lehrach H, Adjaye J (2007) Fibroblast growth factor 2 modulates transforming growth factor beta signaling in mouse embryonic fibroblasts and human ES cells to support hESC self-renewal. Stem Cells 25: 455–464. 17038665 10.1634/stemcells.2006-0476

[6] James D, Levine AJ, Besser D, Hemmati-Brivanlou A (2005) TGFbeta/activin/nodal signaling is necessary for the maintenance of pluripotency in human embryonic stem cells. Development 132: 1273–1282. 15703277 10.1242/dev.01706

[7] Amit M, Shariki C, Margulets V, Itskovitz-Eldor J (2004) Feeder layer- and serum-free culture of human embryonic stem cells. Biol Reprod 70: 837–845. 14627547 10.1095/biolreprod.103.021147

[8] Vallier L, Alexander M, Pedersen RA (2005) Activin/Nodal and FGF pathways cooperate to maintain pluripotency of human embryonic stem cells. J Cell Sci 118: 4495–4509. 16179608 10.1242/jcs.02553

[9] Yu P, Pan G, Yu J, Thomson JA (2011) FGF2 sustains NANOG and switches the outcome of BMP4-induced human embryonic stem cell differentiation. Cell Stem Cell 8: 326–334. 21362572 10.1016/j.stem.2011.01.001 PMC3052735

[10] Bernardo AS, Faial T, Gardner L, Niakan KK, Ortmann D, et al (2011) BRACHYURY and CDX2 mediate BMP-induced differentiation of human and mouse pluripotent stem cells into embryonic and extraembryonic lineages. Cell Stem Cell 9: 144–155. 21816365 10.1016/j.stem.2011.06.015 PMC3567433

[11] Levenstein ME, Ludwig TE, Xu RH, Llanas RA, VanDenHeuvel-Kramer K, et al (2006) Basic fibroblast growth factor support of human embryonic stem cell self-renewal. Stem Cells 24: 568–574. 16282444 10.1634/stemcells.2005-0247 PMC4615709

[12] Liu Y, Song Z, Zhao Y, Qin H, Cai J, et al (2006) A novel chemical-defined medium with bFGF and N2B27 supplements supports undifferentiated growth in human embryonic stem cells. Biochem Biophys Res Commun 346: 131–139. 16753134 10.1016/j.bbrc.2006.05.086

[13] Furue MK, Na J, Jackson JP, Okamoto T, Jones M, et al (2008) Heparin promotes the growth of human embryonic stem cells in a defined serum-free medium. Proc Natl Acad Sci U S A 105: 13409–13414. 18725626 10.1073/pnas.0806136105 PMC2522264

[14] Yao S, Chen S, Clark J, Hao E, Beattie GM, et al (2006) Long-term self-renewal and directed differentiation of human embryonic stem cells in chemically defined conditions. Proc Natl Acad Sci U S A 103: 6907–6912. 16632596 10.1073/pnas.0602280103 PMC1458992

[15] Wang L, Li L, Menendez P, Cerdan C, Bhatia M (2005) Human embryonic stem cells maintained in the absence of mouse embryonic fibroblasts or conditioned media are capable of hematopoietic development. Blood 105: 4598–4603. 15718421 10.1182/blood-2004-10-4065

[16] Xu C, Rosler E, Jiang J, Lebkowski JS, Gold JD, et al (2005) Basic fibroblast growth factor supports undifferentiated human embryonic stem cell growth without conditioned medium. Stem Cells 23: 315–323. 15749926 10.1634/stemcells.2004-0211

[17] Li Y, Powell S, Brunette E, Lebkowski J, Mandalam R (2005) Expansion of human embryonic stem cells in defined serum-free medium devoid of animal-derived products. Biotechnol Bioeng 91: 688–698. 15971228 10.1002/bit.20536

[18] Lu J, Hou R, Booth CJ, Yang SH, Snyder M (2006) Defined culture conditions of human embryonic stem cells. Proc Natl Acad Sci U S A 103: 5688–5693. 16595624 10.1073/pnas.0601383103 PMC1458634

[19] Wang G, Zhang H, Zhao Y, Li J, Cai J, et al (2005) Noggin and bFGF cooperate to maintain the pluripotency of human embryonic stem cells in the absence of feeder layers. Biochem Biophys Res Commun 330: 934–942. 15809086 10.1016/j.bbrc.2005.03.058

[20] Xu RH, Peck RM, Li DS, Feng X, Ludwig T, et al (2005) Basic FGF and suppression of BMP signaling sustain undifferentiated proliferation of human ES cells. Nat Methods 2: 185–190. 15782187 10.1038/nmeth744

[21] Ludwig TE, Levenstein ME, Jones JM, Berggren WT, Mitchen ER, et al (2006) Derivation of human embryonic stem cells in defined conditions. Nat Biotechnol 24: 185–187. 16388305 10.1038/nbt1177

[22] Chen G, Gulbranson DR, Hou Z, Bolin JM, Ruotti V, et al (2011) Chemically defined conditions for human iPSC derivation and culture. Nat Methods 8: 424–429. 21478862 10.1038/nmeth.1593 PMC3084903

[23] Wang L, Schulz TC, Sherrer ES, Dauphin DS, Shin S, et al (2007) Self-renewal of human embryonic stem cells requires insulin-like growth factor-1 receptor and ERBB2 receptor signaling. Blood. 10.1182/blood-2007-03-082586 PMC2190616 17761519

[24] Akopian V, Andrews PW, Beil S, Benvenisty N, Brehm J, et al (2010) Comparison of defined culture systems for feeder cell free propagation of human embryonic stem cells. In Vitro Cell Dev Biol Anim 46: 247–258. 20186512 10.1007/s11626-010-9297-z PMC2855804

[25] Takahashi K, Tanabe K, Ohnuki M, Narita M, Ichisaka T, et al (2007) Induction of pluripotent stem cells from adult human fibroblasts by defined factors. Cell 131: 861–872. 18035408 10.1016/j.cell.2007.11.019

[26] Yu J, Vodyanik MA, Smuga-Otto K, Antosiewicz-Bourget J, Frane JL, et al (2007) Induced pluripotent stem cell lines derived from human somatic cells. Science 318: 1917–1920. 18029452 10.1126/science.1151526

[27] Yu PB, Hong CC, Sachidanandan C, Babitt JL, Deng DY, et al (2008) Dorsomorphin inhibits BMP signals required for embryogenesis and iron metabolism. Nat Chem Biol 4: 33–41. 18026094 10.1038/nchembio.2007.54 PMC2727650

[28] Gerrard L, Rodgers L, Cui W (2005) Differentiation of human embryonic stem cells to neural lineages in adherent culture by blocking bone morphogenetic protein signaling. Stem Cells 23: 1234–1241. 16002783 10.1634/stemcells.2005-0110

[29] Feng XH, Derynck R (2005) Specificity and versatility in tgf-beta signaling through Smads. Annu Rev Cell Dev Biol 21: 659–693. 16212511 10.1146/annurev.cellbio.21.022404.142018

[30] D'Amour KA, Agulnick AD, Eliazer S, Kelly OG, Kroon E, et al (2005) Efficient differentiation of human embryonic stem cells to definitive endoderm. Nat Biotechnol 23: 1534–1541. 16258519 10.1038/nbt1163

[31] Chen B, Dodge ME, Tang W, Lu J, Ma Z, et al (2009) Small molecule-mediated disruption of Wnt-dependent signaling in tissue regeneration and cancer. Nat Chem Biol 5: 100–107. 19125156 10.1038/nchembio.137 PMC2628455

[32] Greber B, Lehrach H, Adjaye J (2008) Control of early fate decisions in human ES cells by distinct states of TGFbeta pathway activity. Stem Cells Dev 17: 1065–1077. 18393632 10.1089/scd.2008.0035

[33] Chambers SM, Fasano CA, Papapetrou EP, Tomishima M, Sadelain M, et al (2009) Highly efficient neural conversion of human ES and iPS cells by dual inhibition of SMAD signaling. Nat Biotechnol 27: 275–280. 19252484 10.1038/nbt.1529 PMC2756723

[34] Cowan CA, Klimanskaya I, McMahon J, Atienza J, Witmyer J, et al (2004) Derivation of embryonic stem-cell lines from human blastocysts. N Engl J Med 350: 1353–1356. 14999088 10.1056/NEJMsr040330

[35] Zhang X, Stojkovic P, Przyborski S, Cooke M, Armstrong L, et al (2006) Derivation of human embryonic stem cells from developing and arrested embryos. Stem Cells 24: 2669–2676. 16990582 10.1634/stemcells.2006-0377

[36] Greber B, Coulon P, Zhang M, Moritz S, Frank S, et al (2011) FGF signalling inhibits neural induction in human embryonic stem cells. EMBO J 30: 4874–4884. 22085933 10.1038/emboj.2011.407 PMC3243624

[37] Passier R, Oostwaard DW, Snapper J, Kloots J, Hassink RJ, et al (2005) Increased cardiomyocyte differentiation from human embryonic stem cells in serum-free cultures. Stem Cells 23: 772–780. 15917473 10.1634/stemcells.2004-0184

[38] Greber B, Wu G, Bernemann C, Joo JY, Han DW, et al (2010) Conserved and divergent roles of FGF signaling in mouse epiblast stem cells and human embryonic stem cells. Cell Stem Cell 6: 215–226. 20207225 10.1016/j.stem.2010.01.003

[39] Huangfu D, Maehr R, Guo W, Eijkelenboom A, Snitow M, et al (2008) Induction of pluripotent stem cells by defined factors is greatly improved by small-molecule compounds. Nat Biotechnol 26: 795–797. 18568017 10.1038/nbt1418 PMC6334647

[40] Moretti A, Bellin M, Welling A, Jung CB, Lam JT, et al (2010) Patient-specific induced pluripotent stem-cell models for long-QT syndrome. N Engl J Med 363: 1397–1409. 20660394 10.1056/NEJMoa0908679

