# Supplementary figures and images for: Small Molecule-Assisted, Line-Independent Maintenance of Human Pluripotent Stem Cells in Defined Conditions
Source: PLoS One. 2012 Jul 30;7(7):e41958. doi: 10.1371/journal.pone.0041958 (PMC3408405; doi:10.1371/journal.pone.0041958)

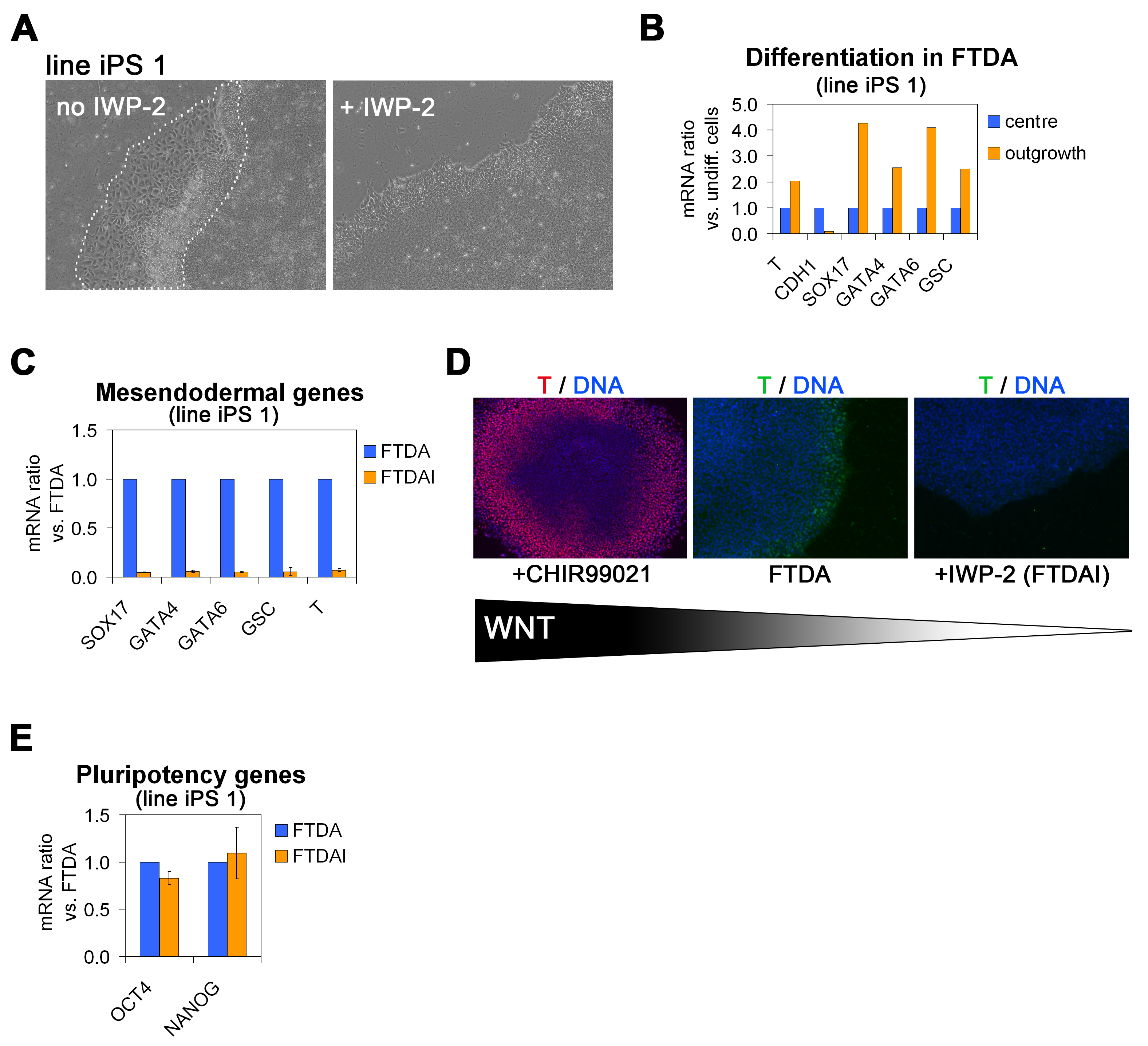

Supplement: Figure S1 — Activation of WNT signaling is counterproductive for hESC self-renewal. (A) Human iPS line 1 showed distinct outgrowths of differentiating cells when cultured in FTDA (left). Application of 2 µM IWP-2 completely blocked this differentiation (right). Representative phase contrast morphology. (B) Gene expression analysis of FTDA-mediated outgrowth of cells in iPS line 1. Outgrowing cells showed upregulation of mesendodermal markers, whereas the epithelial cell marker CDH1 was downregulated compared to the undifferentiated colony centers. (C) Selective inhibition of endogenous WNT signaling by adding IWP-2 to FTDA (FTDAI) resulted in strong downregulation of mesendodermal markers (n = 3, iPS line 1). (D) Brachyury (T) expression in hESCs depended on WNT signaling. Left: Activation of canonical WNT signaling by continuous exposure to GSK3β inhibitor CHIR99021 (3 µM) induced T expression at the edges of hESC colonies. Middle: In normal FTDA culture, only a small number of T-positive cells could be found at the edges of hESC colonies. Right: Small molecule-inhibition of endogenous WNT signaling (IWP-2) completely removed T-positve cells at the colony periphery. (E) IWP-2 had no effect on expression levels of pluripotency markers OCT4 and NANOG (n = 3, iPS line 1). (TIF) [file pone.0041958.s001.tif]

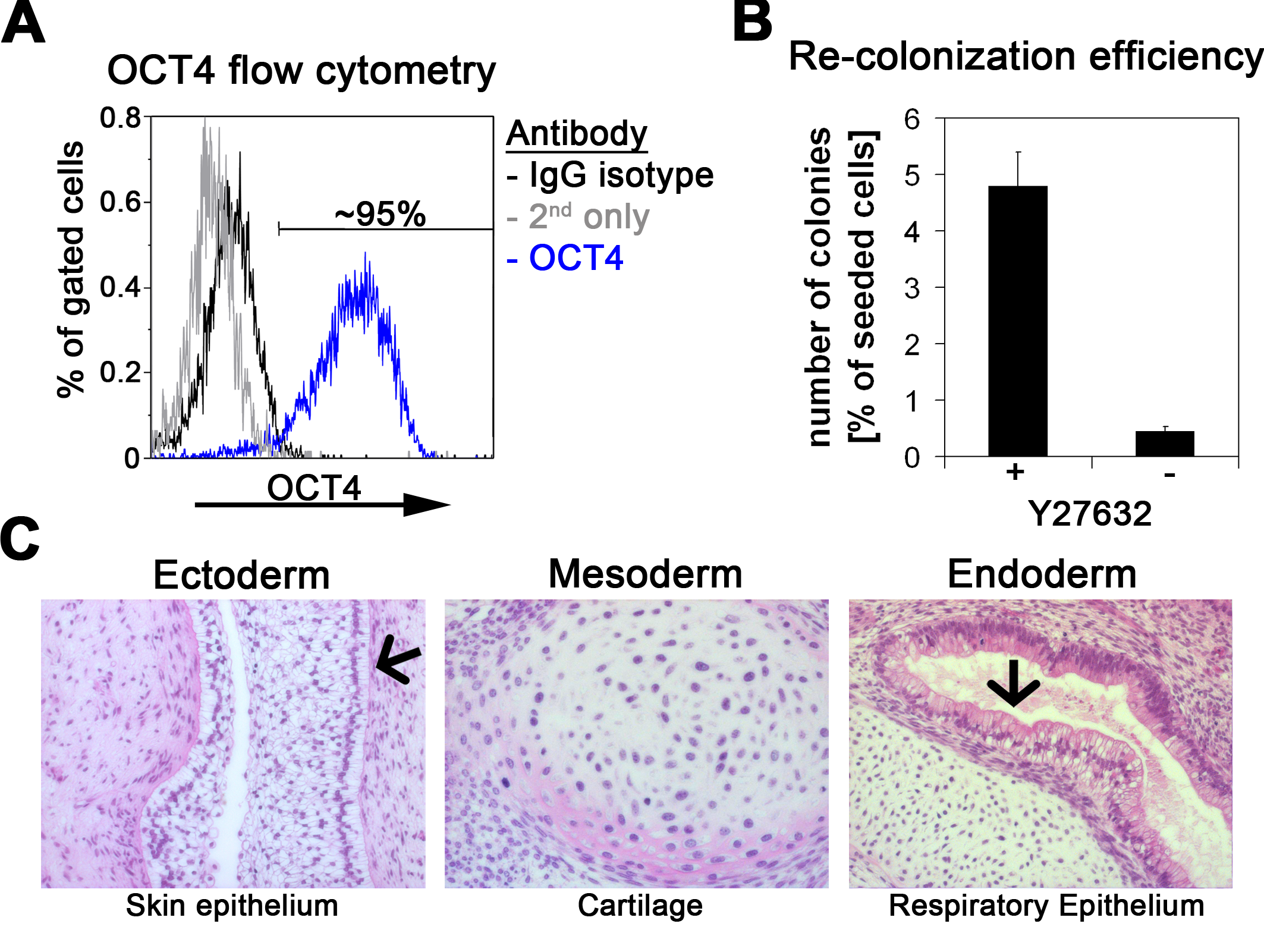

Supplement: Figure S2 — Long-term maintenance of pluripotency features in FTDA. (A) Flow cytometry of NCL3 hESCs cultured in FTDA revealed 95% OCT4-positive cells. (B) Single cell-plating of hESCs in FTDA required addition of 10 µM ROCK-inhibitor Y27632. Plating efficiency was ∼5% (of all plated hESCs, n = 3). (C) H&E stained teratoma sections formed by HuES6 cells grown for more than 20 passages in the defined medium. (TIF) [file pone.0041958.s002.tif]
